# Higher levels of the soluble receptor for advanced glycation end products and lower levels of the extracellular newly identified receptor for advanced glycation end products were associated with lipid-lowering drugs in patients with type 1 diabetes: a comparative cross-sectional study

**DOI:** 10.1186/s12944-020-01397-2

**Published:** 2020-10-14

**Authors:** Eva O. Melin, Jonatan Dereke, Magnus Hillman

**Affiliations:** 1 Faculty of Medicine, Department of Clinical Sciences, Diabetes Research Laboratory, Lund University, Lund, Sweden; 2 Department of Research and Development, Region Kronoberg, Box 1223, SE-35112 Växjö, Sweden

**Keywords:** Atherosclerosis, Extracellular newly identified receptor for advanced glycation end products (EN-RAGE), Inflammation, Soluble receptor for advanced glycation end products (sRAGE), Type 1 diabetes

## Abstract

**Background:**

The receptors for advanced glycation end products (RAGE) are increased in atherosclerotic plaques. Soluble (s)RAGE decreases, whereas the extracellular newly identified receptor for advanced glycation end products (EN-RAGE) increases inflammatory responses mediated by RAGE. The aims were to explore whether sRAGE, EN-RAGE and the EN-RAGE/sRAGE ratio, were associated with the use of lipid-lowering drugs (LLD) and/or antihypertensive drugs (AHD) in patients with type 1 diabetes (T1D).

**Methods:**

Cross-sectional design. T1D patients were consecutively recruited from one diabetes clinic. Blood samples were collected, supplemented with data from electronic health records. sRAGE and EN-RAGE were analysed by enzyme linked immunosorbent assays. An EN-RAGE/sRAGE ratio was calculated. Adjustments were performed with inflammatory and metabolic variables, s-creatinine, depression, smoking, physical inactivity, medication, and cardiovascular complications. Multiple regression analyses were performed.

**Results:**

In this study 283 T1D patients (men 56%, 18–59 years) were included. One-hundred and thirty LLD users compared to 153 non-users had lower levels of the EN-RAGE/sRAGE ratio (*P* = 0.009), and 89 AHD users compared to 194 non-users had lower levels of sRAGE (*P* = 0.031). The use of LLD (inversely) (B coefficient − 0.158, *P* = 0.033) and the use of AHD (B coefficient 0.187, *P* = 0.023) were associated with the EN-RAGE/sRAGE ratio. sRAGE (Lg10) (per unit) (adjusted odds ratio (AOR) = 3.5, 95% CI = 1.4–9.1, *P* = 0.009), EN-RAGE (Lg10) (per unit) (inversely) (AOR 0.4, 95% CI = 0.2–1.0, *P =* 0.046), age (*P* <  0.001), and triglycerides (*P* <  0.029), were associated with LLD. sRAGE (Lg10) (per unit) (inversely) (AOR = 0.2, 95% CI = 0.1–0.5, *P* = 0.001), diabetes duration, triglycerides, s-creatinine, and systolic BP (all *P* values < 0.043), were associated with AHD.

**Conclusions:**

Higher sRAGE levels and lower EN-RAGE levels were linked to the use of LLD, whereas lower sRAGE levels were linked to the use of AHD. No other variables but the use of LLD and the use of AHD were linked to the EN-RAGE/sRAGE ratio. This may be of major importance as sRAGE is an inhibitor and EN-RAGE is a stimulator of inflammatory processes mediated by RAGE.

## Background

Type 1 diabetes (T1D) is an autoimmune disease, characterized by insulin deficiency leading to hyperglycemia [1]. T1D is linked to increased risk for myocardial infarction, heart failure, and ischemic stroke [2], and an almost threefold higher all-cause mortality rate than in the general population, primarily due to atherosclerosis [3, 4]. Traditional risk factors such as hyperglycemia, hypertension, and dyslipidaemia, as well as the production and accumulation of advanced glycation end products (AGEs), and complex inflammatory reactions, contribute to the development of atherosclerosis and cardiovascular complications [3–9].

AGEs are late-stage glycoxidation and glycation adducts of proteins and lipids that accumulate at an increased rate in the plasma and tissues of patients with diabetes due to increased levels of glucose, superimposed oxidant stress, and inflammatory disturbances [4, 10]. The receptor for advanced glycation end products (RAGE) is a multiligand member of the immunoglobulin superfamily [4, 6, 8, 11]. In addition to AGEs, RAGE binds certain members of the S100/calgranulin family, high-mobility group box 1 (HMGB1), β-amyloid peptide, and β-sheet fibrils [4, 8, 11]. The interaction of RAGE with its ligands upregulates RAGE itself, and several proinflammatory molecules such as TNF-α, IL-6, and IL-1β [4, 12]. The activation of RAGE by AGEs increases accumulation of lipids in macrophages [13], and promotes complex vascular lesions [4]. Beneficial effects of RAGE antagonism have been demonstrated [8, 11]. Different soluble isoforms of RAGE (sRAGE) circulate in plasma [12]. The total amount of circulating sRAGE includes cleaved RAGE and endogenous secretory RAGE (es-RAGE) [12]. sRAGE acts as a decoy for ligands, and blocks their interaction with RAGE and thus prevents inflammatory responses mediated by RAGE [8, 12, 14]. Administration of sRAGE to diabetic mice showed decreased vascular inflammation and stabilized atherosclerotic lesions [6, 8, 15]. In humans it has been shown that higher levels of plasma sRAGE were associated with lower carotid intima-media thickness progression, and lower risk for first-time coronary events [16]. Lower levels of sRAGE have been observed in patients with essential hypertension, and sRAGE was inversely related to pulse pressure [17]. The extracellular newly identified receptor for advanced glycation end products (EN-RAGE), also named S100A12, is a member of the S100/calgranulin family of proinflammatory cytokines [7, 8, 11]. EN-RAGE serves as a ligand of RAGE [11], and is involved in chronic inflammation in the atherosclerotic lesions [10]. EN-RAGE induces adhesion molecules in the vascular endothelial cell, and mediates migration and activation of monocytes and macrophages through RAGE binding [10]. Higher levels of EN-RAGE have been demonstrated in patients with T1D [18], and higher levels of EN-RAGE were correlated with higher HbA1c both in patients with type 2 diabetes (T2D), and in non-diabetic subjects [10]. Statins are lipid-lowering, anti-inflammatory, and anti-atherosclerotic medications, with beneficial impact on cardiovascular morbidity and mortality [9]. In a study of patients with hypercholesterolemia it was demonstrated that sRAGE levels were higher in the patients who were treated with a statin [14]. RAGE expression was inhibited by a statin in human atherosclerotic lesions in a glucose-independent manner [19]. In an in vitro study of monocytes, it was shown that up-regulation of RAGE and EN-RAGE by the pro-inflammatory cytokine C-reactive protein (CRP) was inhibited by a statin [20]. sRAGE was increased in patients treated with an angiotensin-converting enzyme (ACE) inhibitor [21]. Both sRAGE and RAGE were decreased in patients treated with angiotensin II receptor blockers (ARB) [22, 23].

None of these described studies explored the use of lipid-lowering drugs (LLD) or antihypertensive drugs (AHD) in relation to the RAGE, EN-RAGE, and sRAGE system in a setting of T1D patients [14, 19–23]. The hypotheses were that the use of lipid-lowering drugs (LLD) and antihypertensive drugs (AHD) may have impact on the levels of EN-RAGE and sRAGE in patients with T1D. The aims were to explore whether EN-RAGE, sRAGE, and a ratio between EN-RAGE and sRAGE, were associated with the use of LLD and AHD in a setting of adult patients with T1D.

## Methods

**Fig. 1:**
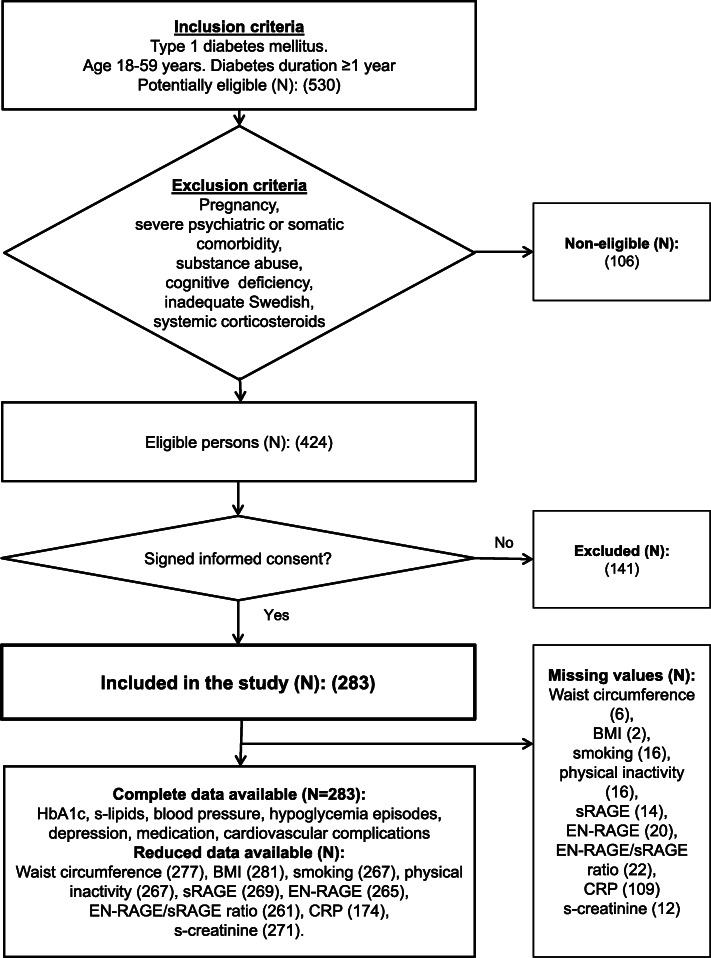
Flow chart showing inclusion and exclusion criteria, and missing values

The study has a cross sectional design, and is one of several studies addressing metabolic and inflammatory disturbances in a cohort of patients with T1D [24–30]. For inclusion and exclusion criteria, number of included and excluded patients, and missing variables, see Fig. 1. Inclusion criteria were T1D with ≥1-year duration in patients 18–59 years of age. Exclusion criteria were pregnancy, severe somatic and psychiatric disorders such as cancer, hepatic failure, end-stage renal disease (ESRD), Cushing’s disease, severe autoimmune disorders such as systemic lupus erythematosus, psychotic disorders, bipolar disorder, severe personality disorders, severe substance abuse, cognitive deficiency (due to stroke, dementia or mental retardation), or inadequate knowledge of Swedish. As patients with these disorders were excluded, no specific medications for any of these disorders were used by the included patients. Nine patients using systemic corticosteroids were excluded as the use of systemic corticosteroids were associated with AHD and the EN-RAGE/sRAGE ratio. Patients using other types of medication were not excluded.

The patients were recruited from the largest of two specialist diabetes hospital outpatient clinics where adult patients with T1D are treated in Region Kronoberg, Sweden. The catchment population was 125,000. The patients who attended the clinic every six months for regular follow up visits were consecutively recruited by specialist diabetes physicians or diabetes nurses during a nine-month period, 25 March 2009 to 28 December 2009 [24–26, 28–30]. Blood samples, anthropometrics, and blood pressure (BP) were collected, and supplemented with data from electronic health records. An EN-RAGE/sRAGE ratio was calculated. Adjustments were performed with age, diabetes duration, sex, plasma C-reactive protein (p-CRP), total cholesterol, low-density lipoprotein (LDL)-cholesterol, high-density lipoprotein (HDL)-cholesterol, triglycerides, severe hypoglycemia episodes, s-creatinine, depression smoking, physical inactivity, medication (continuous subcutaneous insulin infusion (CSII), AHD, LLD, antidepressants, nasal and inhaled steroids), and cardiovascular complications.

### Biochemical analyses

sRAGE and EN-RAGE were analysed using commercial human DuoSet enzyme linked immunosorbent assays (ELISAs) and supplementary ancillary kit (R&D Systems, Minneapolis, MN, USA). Patient plasma samples were diluted 1:5 or 1:300 for sRAGE and EN-RAGE respectively in phosphate-buffered saline (PBS) supplemented with 1% bovine serum albumin (BSA) and run in duplicates. The ELISA analyses were performed according to the manufacturer’s instructions. Absorbance was measured at 450–580 nm in a FLOUstar optima plate reader (BMG Labtech Gmbh, Ortenberg, Germany). Concentrations of unknown samples were calculated using a 4-parameter logistic regression curve. The intra-assay coefficient of variation was 6.3 and 3.6% respectively for sRAGE and EN-RAGE. sRAGE and EN-RAGE were analysed at the Diabetes Laboratory, BMC, Lund University, Lund.

P-CRP was analysed with a Roche Cobas C501® analyser (Basel, Switzerland). The measurement is based on a particle enhanced immunoturbidimetric analysis where p-CRP agglutinates with latex particles covered with monoclonal anti-human-CRP antibodies. Absorbance is measured at 570 and 800 nm and is directly proportional to the CRP-concentration. The intra-assay coefficient of variation was < 5.5%. P-CRP were analysed at the department of clinical chemistry, Lund University Hospital.

HbA1c and serum (s)-lipids were collected after overnight fasting and analysed with an Olympus automated clinical chemistry analyser with high specificity (Olympus AU®, Tokyo, Japan). The intra-coefficients of variation were for HbA1c < 1.2%; total cholesterol < 2.1%; HDL-cholesterol < 3.0%; LDL-cholesterol < 2,6%; and for triglycerides < 2.2%.

S-creatinine was assayed by an AU2700® instrument (Beckman Coulter, Brea, Ca, USA). The intra-coefficient of variation was < 3%.

HbA1c, s-lipids and s-creatinine were analysed at the department of Clinical Chemistry, Växjö Central Hospital.

### Anthropometrics and blood pressure

Waist circumference (WC), weight, length, and BP, were measured according to standard procedures by a nurse. Abdominal obesity was defined as WC ≥1.02 m for men and as WC ≥0.88 m for women [25, 31]. General obesity was defined as BMI ≥30 kg/m^2^ for both sexes [25, 31].

### Episodes of hypoglycemia

A severe episode of hypoglycemia was defined as needing help from another person. Episodes during the last 6 months prior to recruitment were registered.

### Self-reported depression

Self-reported depression was assessed by the Hospital Anxiety and Depression Scale-Depression subscale (HADS-D) which consists of 7 statements, with 4 response alternatives from 0 to 3. Depression was defined as HADS-D ≥ 8 points as in previous research in these patients [24, 26, 28, 32].

### Smoking and physical inactivity

Smokers were defined as having smoked any amount of tobacco during the last year. Levels of physical activity were assessed by interviews performed by skilled nurses and physicians at the regular follow-up visits. Levels of physical activity performed at work and during leisure time were evaluated. Physical activity was dichotomized into physical inactivity which was defined as less than 30 min of moderate activities once a week, and physical activity which represents all other levels of physical activity [24].

### Medication

Patients used either multiple daily insulin injections (MDII) or CSII.

LLD were only hydroxy-methylglutaryl coenzyme A (HMG-CoA) reductase inhibitors (statins) (ATC-code C10AA). Indications for LLD were total cholesterol > 4.5 mmol/L (> 1.74 mg/dL) and/or LDL-cholesterol > 2.5 mmol/L (> 97 mg/dL) or present cardiovascular complications according to the Swedish national guidelines in 2009 [33]. The use of LLD was dichotomized into users and non-users as in previous research in these patients [25, 26].

AHD included ACE inhibitors (ATC codes C09AA-BA); (ARB) (ATC codes C09CA-DA); calcium antagonists (ATC codes C08CA01–02); diuretics (ATC codes C03AA03 or C03CA01); and/or selective beta-adrenoreceptor antagonists (ATC code C07AB). Indications for AHD were systolic BP > 130 mmHg and/or BP > 80 mmHg and/or cardiovascular complications according to the Swedish national guidelines in 2009 [33]. The use of AHD was dichotomized into users and non-users as in previous research in these patients [25].

Corticosteroids were nasal corticosteroids (ATC code R01AD) and inhaled corticosteroids (ATC code R03BA).

Antidepressants were SSRIs, SNRIs and/or specific serotonergic antidepressants (N06AB, N06AX16, or N06AX11).

### Cardiovascular complications

Cardiovascular complications were defined as ischemic heart disease, cardiac failure, stroke, or transient ischemic attack [25].

### Statistical analysis

Analysis of data distribution using histograms revealed that EN-RAGE, sRAGE, EN-RAGE/sRAGE ratio, p-CRP, total cholesterol, HDL-cholesterol, triglycerides, and s-creatinine, were not normally distributed. Data were presented as median (quartile (q)_1_, q_3_) and the analyses were performed with Mann-Whitney *U* test. Fisher’s Exact Test (two-tailed) was used to analyse categorical data, and data were presented as N (%).

sRAGE, EN-RAGE, the EN-RAGE/sRAGE ratio, p-CRP, total cholesterol, HDL-cholesterol, triglycerides, and s-creatinine were log transformed. Simple and multiple linear regression analyses were performed with EN-RAGE (Lg10), sRAGE (Lg10) and EN-RAGE/sRAGE (Lg10) as dependent variables. Variables with *P* ≤ 0.20 in the simple linear regression analyses were entered into multiple linear regression analyses.

Crude odds ratios (CORs) were calculated for associations with “use of LLD” and with “use of AHD” as dependent variables for all included variables. Variables with *P* ≤ 0.20 for the CORs were entered into multiple logistic regression analyses (Backward: Wald) with “use of LLD” and “use of AHD” as dependent variables in two separate models for each dependent variable. In the first model, EN-RAGE (Lg10) and sRAGE (Lg10) were included. In the second model, EN-RAGE and sRAGE were replaced by the EN-RAGE/sRAGE ratio (Lg10). The Hosmer and Lemeshow test for goodness-of-fit and Nagelkerke R^2^ were used to evaluate each multiple logistic regression analysis model. Confidence intervals (CIs) of 95% were used.

*P* <  0.05 was considered statistically significant. SPSS® version 23 (IBM, Chicago, Il, USA) was used.

## Results

In this study 283 patients with T1D (men 56%, 18–59 years old, diabetes duration 1–55 years, users of LLD 46%, users of AHD 31%) were included. Fifty-eight patients (20%) used both LLD and AHD. In Table 1, baseline characteristics are compared between 130 users and 53 non-users of LLD, and between 89 users and 194 non-users of AHD. Both the users of LLD and the users of AHD compared to the non-users of these drugs were older, had longer diabetes duration, higher systolic BP (all *P* <  0.001), and had higher prevalence of cardiovascular complications (*P* = 0.007 and *P* = 0.013 respectively). The users of LLD compared to the non-users of LLD had higher prevalence of abdominal obesity (*P* = 0.015). The users of AHD compared to the non-users of AHD had higher diastolic BP (*P* = 0.007).

**Table 1:** Baseline characteristics compared between users and non-users of LLD and AHD

	All patients ^**a**^	Users of lipid-lowering drugs ^**b**^			Users of antihypertensive drugs		
		Yes	No	*P* value ^c^	Yes	No	*P* value ^c^
*N*	283	130	153	–	89	194	–
Age, years	(18–59)	48 (42, 54)	34 (27, 44)	< 0.001	49 (42, 56)	38 (29, 47)	< 0.001
Diabetes duration, years	(1–55)	26 (14, 35)	17 (9, 23)	< 0.001	29 (20, 36)	16 (9, 25)	< 0.001
Sex							
Women	124 (44)	54 (42)	70 (46)	0.55	31 (35)	93 (48)	0.040
Men	159 (56)	76 (58)	83 (54)		58 (65)	101 (52)	
Abdominal obesity ^e^	46 (16)	29 (23)	17 (11)	0.015 ^d^	20 (23)	26 (14)	0.082
General obesity ^f^	34 (12)	16 (12)	18 (12)	> 0.99 ^d^	10 (11)	24 (12)	0.85
Hypoglycemia, severe episodes	12 (4)	7 (5)	5 (3)	0.39 ^d^	2 (2)	10 (5)	0.35
Systolic BP, mmHg	(90–160)	125 (119, 135)	120 (110, 130)	< 0.001	130 (125, 138)	120 (110, 125)	< 0.001
Diastolic BP, mmHg	(55–100)	70 (70, 75)	70 (65, 75)	0.44	70 (70, 79)	70 (65, 75)	0.007
Depression	29 (10)	14 (11)	15 (10)	0.84	10 (11)	19 (10)	0.68
Smoking ^g^	28 (10)	13 (10)	15 (11)	> 0.99 ^d^	6 (7)	22 (12)	0.28
Physical inactivity ^h^	29 (11)	10 (8)	19 (14)	0.17 ^d^	8 (9)	21 (12)	0.68
Continuous subcutaneous insulin infusion	25 (9)	10 (8)	15 (10)	0.68^d^	7 (8)	18 (9)	0.82
Lipid-lowering drugs	130 (46)	–	–		58 (65)	72 (37)	< 0.001
Antihypertensive drugs	89 (31)	58 (45)	31 (20)	< 0.001 ^d^	–	–	–
Antidepressants	22 (8)	15 (12)	7 (5)	0.043	9 (10)	13 (7)	0.34
Corticosteroids, nasal or inhaled	26 (9)	13 (10)	13 (8)	0.68	10 (11)	16 (8)	0.51
Cardiovascular complications	10 (4)	9 (7)	1 (1)	0.007 ^d^	7 (8)	3 (2)	0.013

^a^ Data are presented as (min-max) or N (%). ^b^ Data are presented as N (%) or median (q_1_, q_3_)

^c^ Mann-Whitney *U* test unless otherwise indicated. ^d^ Fisher’s exact test. Missing values (N): ^e^ 6; ^f^ 2; ^g, h^ 16

In Table 2, results from the biochemical analyses are compared between the users and the non-users of LLD, and between the users and the non-users of AHD. The users of LLD compared to the non-users of LLD had lower levels of the EN-RAGE/sRAGE ratio (*P* = 0.009) and higher levels of HbA1c (*P* = 0.022). The users of AHD compared to the non-users of AHD had lower levels of sRAGE (*P* = 0.031) and higher levels of s-creatinine (*P* = 0.022). Both the users of LLD and the users of AHD compared to the non-users of these drugs had higher levels of triglycerides (*P* = 0.016 and *P* = 0.043 respectively).

**Table 2:** Biochemical analyses compared between users and non-users of LLD and AHD

	All patients ^**a**^	Users of lipid-lowering drugs ^**b**^			Users of antihypertensive drugs		
		Yes	No	*P* value ^c^	Yes	No	*P* value ^c^
*N*	283	130	153	–	89	194	–
EN-RAGE ^d^, μg/L	(1.5–907)	11.8 (6.8, 18.8)	13.7 (8.0, 24.9)	0.060	12.8 (7.0, 18.9)	13.2 (7.4, 23.4)	0.48
sRAGE ^e^, μg/L	(0.2–105)	1.3 (0.8, 2.0)	1.3 (0.8, 1.8)	0.38	1.1 (0.6, 1.7)	1.3 (0.9, 1.9)	0.031
EN-RAGE/sRAGE ratio ^f^	(0.4–756)	7.9 (4.0, 17.7)	11.7 (6.7, 22.8)	0.009	10.8 (4.5, 29.4)	10.0 (5.3, 17.4)	0.19
CRP ^g^, mg/L	(0.03–8.9)	0.5 (0.2, 1.7)	0.8 (0.3, 1.6)	0.67	0.6 (0.3, 1.7)	0.7 (0.3, 1.7)	0.86
HbA1c							
mmol/mol	(25–110)	64 (56, 72)	61 (53, 69)	0.022	65 (55, 72)	60 (54, 71)	0.090
%	(4.4–12.2)	8.0 (7.3, 8.7)	7.8 (7.0, 8.5)		8.1 (7.2, 8.8)	7.7 (7.1, 8.6)	
Total cholesterol, mmol/L	(2.1–10.9)	4.5 (4.0, 5.2)	4.6 (4.1, 5.2)	0.37	4.7 (4.0, 5.3)	4.6 (4.1, 5.1)	0.69
Triglycerides, mmol/L	(0.1–5.9)	1.0 (0.7, 1.3)	0.8 (0.7, 1.1)	0.016	1.0 (0.7, 1.4)	0.9 (0.6, 1.2)	0.043
LDL-cholesterol, mmol/L	(0.6–8.3)	2.7 (2.3, 3.3)	2.9 (2.4, 3.3)	0.12	2.8 (2.3, 3.4)	2.8 (2.4, 3.3)	0.98
HDL-cholesterol, mmol/L	(0.3–2.7)	1.5 (1.3, 1.8)	1.5 (1.3, 1.8)	0.52	1.5 (1.3, 1.8)	1.5 (1.3, 1.8)	0.60
S-creatinine ^h^_,_ μmol/L	(28–182)	71 (64, 80)	69 (61, 76)	0.088	72 (63, 81)	69 (61, 77)	0.022

^a^ Data are presented as (min-max). ^b^ Data are presented as median (q_1_, q_3_). ^c^ Mann-Whitney *U* test unless indicated

Missing values (N): ^d^ 18; ^e^ 14; ^f^ 22; ^g^ 109 ^h^ 12

The association with EN-RAGE (Lg10) used as the dependent variable was significant for p-CRP (B coefficient = 0.190, *P* = 0.002), but not for sRAGE (Lg10) (B coefficient = 0.160, *P* = 0.085), or any other variables included in the study (all *P* values > 0.14). The associations with sRAGE used as the dependent variable were significant for AHD (Lg10) (inversely) (B coefficient = − 0.200, *P* = 0.001) and s-creatinine (B coefficient = 0.587, *P* = 0.044), but not for EN-RAGE (Lg10) (B coefficient = 132, *P* = 0.059), or the use of LLD (B coefficient = 0.100, *P* = 0.065), or any other variables included in the study (all *P* values > 0.24). The associations with the EN-RAGE/sRAGE ratio (Lg10) used as the dependent variable were significant for the use of AHD (B coefficient 0.187, *P* = 0.023) and the use of LLD (inversely) (B coefficient − 0.158, *P* = 0.033), but not for any other variables included in the study (all *P* values > 0.14).

In Table 3 variables associated with the use of LLD are presented. In model 1, age (per year) (adjusted odds ratio (AOR) = 1.12, 95% CI = 1.08–1.16, *P* <  0.001), EN-RAGE (Lg10) (per unit) (AOR = 0.4, 95% CI = 0.2–1.0, *P =* 0.046), sRAGE (Lg10) (per unit) (AOR = 3.5, 95% CI = 1.4–9.1, *P* = 0.009), and triglycerides (Lg10) (per unit) (AOR = 3.5, 95% CI = 1.4–9.1, *P* = 0.029), were associated with the use of LLD. In model 2, the EN-RAGE/sRAGE ratio (Lg10) (AOR = 0.3, CI = 0.2–0.7, *P* = 0.003) was inversely associated with the use of LLD.

**Table 3:** Associations with LLD presented for two separate models

	Use of lipid-lowering drugs					
	COR (95% CI)	*P* value	Model 1		Model 2	
			AOR (95% CI)	*P* value ^*a*^	AOR (95% CI)	*P* value ^*b*^
Age, per year	1.11 (1.08–1.14)	< 0.001	1.12 (1.08–1.16)	< 0.001	1.12 (1.08–1.16)	< 0.001
Diabetes duration, per year	1.05 (1.03–1.07)	< 0.001	1.00 (0.97–1.03)	0.94	1.00 (0.97–1.03)	0.85
Sex, women	0.8 (0.5–1.4)	0.48	–	–	–	–
EN-RAGE, Lg10, per unit	0.5 (0.3–1.0)	0.059	0.4 (0.2–1.0)	0.046	–	–
sRAGE, Lg10, per unit	1.7 (0.9–3.6)	0.13	3.5 (1.4, 9.1)	0.009	–	–
EN-RAGE/sRAGE ratio, Lg10, per unit	0.5 (0.3–0.8)	0.010	–	–	0.3 (0.2–0.7)	0.003
CRP, Lg10, per unit	1.0 (0.5–1.7)	0.87	–	–	–	–
HbA1c, per mmol/mol	1.02 (1.00–1.04)	0.026	1.01 (0.99–1.04)	0.34	1.01 (0.99–1.04	0.34
Total cholesterol, Lg10, per unit	0.8 (0.1–12.4)	0.89	–	–	–	–
Triglycerides, Lg10, per unit	3.2 (1.1–9.5)	0.038	3.5 (1.4–9.1)	0.029	5.0 (1.2–21.3)	0.030
LDL-cholesterol, per mmol/L	0.9 (0.7–1.3)	0.72	–	–	–	–
HDL-cholesterol, Lg10, per unit	1.3 (0.2–11.5)	0.79	–	–	–	–
S-creatinine, Lg10, per unit	11.4 (0.9–150)	0.064	0.8 (0.02–36)	0.92	1.2 (0.03–47)	0.93
Abdominal obesity	2.3 (1.2–4.4)	0.014	2.1 (0.8–5.1)	0.11	2.0 (0.8–4.9)	0.12
Hypoglycemia, severe episodes	1.7 (0.5–5.5)	0.38	–	–	–	–
Systolic BP, per mm Hg	1.05 (1.02–1.07)	< 0.001	0.99 (0.96–1.02)	0.55	0.99 (0.96–1.02)	0.56
Diastolic BP, per mm Hg	1.02 (0.98–1.05)	0.38	–	–	–	–
Depression	1.1 (0.4–2.4)	0.79	–	–	–	–
Smoking	1.0 (0.4–2.2)	0.96	–	–	–	–
Physical inactivity	0.6 (0.2–1.2)	0.15	0.9 (0.3–3.0)	0.93	1.0 (0.3–3.1)	0.94
Continuous subcutaneous insulin infusion	0.8 (0.3–1.8)	0.53	–	–	–	–
Antihypertensive drugs	3.2 (1.9–5.4)	< 0.001	1.6 (0.8–3.2)	0.19	1.6 (0.8–3.1)	0.21
Antidepressants	2.7 (1.1–6.9)	0.035	1.3 (0.4–4.5)	0.64	1.4 (0.4–4.7)	0.59
Corticosteroids, nasal or inhaled	1.2 (0.5–2.7)	0.66	–	–	–	–
Cardiovascular complications	11.3 (1.4–90	0.022	1.3 (0.1–12.5)	0.82	1.4 (0.2–13.4)	0.75

^a, b^ Multiple regression analyses (Backward: Wald): N = ^a, b^ 239; Nagelkerke R Square ^a^ 0.391/^b^ 0.389

Hosmer and Lemeshow Test ^a^ 0.024/^b^ 0.010. For missing values, see Tables 1 and 2

In Table 4 variables associated with the use of AHD are presented. In model 1 diabetes duration (per year) (AOR = 1.09, 95% CI = 1.09–1.12, *P* <  0.001), sRAGE (Lg10) (per unit) (inversely) (AOR = 0.2, 95% CI = 0.01–0.5, *P* = 0.001), triglycerides (Lg10) (per unit) (AOR = 7.2, 95% = 1.4–37, *P* = 0.019), s-creatinine (AOR = 52.1, 95% CI = 1.1–2386, *P* = 0.043), and systolic BP (AOR = 1.07, 95% CI = 1.04 – 1.11, *P* <  0.001), were associated with the use of AHD.

In model 2, the EN-RAGE/sRAGE ratio (Lg10) (per unit) (AOR = 1.7, 95% CI = 0.9–3.4, *P* = 0.13) was not associated with the use of AHD.

**Table 4:** Associations with AHD presented for two separate models

	Use of antihypertensive drugs					
	COR (95% CI)	*P* value	Model 1		Model 2	
			AOR (95% CI)	*P* value ^*a*^	AOR (95% CI)	*P* value ^*b*^
Age, per year	1.08 (1.05–1.11)	< 0.001	1.02 (0.98–1.06)	0.44	1.01 (0.97–1.05)	0.62
Diabetes duration, per year	1.08 (1.06–1.11)	< 0.001	1.09 (1.06–1.12)	< 0.001	1.08 (1.05–1.12)	< 0.001
Sex, women	0.6 (0.3–1.0)	0.040	1.0 (0.4–2.2)	0.95	0.9 (0.4–2.1)	0.84
EN-RAGE, Lg10, per unit	0.7 (0.4–1.5)	0.41	–	–	–	–
sRAGE, Lg10, per unit	0.4 (1.2–0.9)	0.018	0.2 (0.1–0.5)	0.001	–	–
EN-RAGE/sRAGE, Lg10, per unit	1.5 (0.8–2.7)	0.17	–	–	1.7 (0.9–3.4)	0.13
CRP, Lg10, per unit	1.1 (0.6–2.1)	0.75	–	–	–	–
HbA1c, per mmol/mol	1.01 (1.00–1.03)	0.13	0.99 (0.96–1.02)	0.65	0.99 (0.96–1.02)	0.58
Total cholesterol, Lg10, per unit	2.3 (0.1–41)	0.58	–	–	–	–
Triglycerides, Lg10, per unit	4.5 (1.4–14.5)	0.011	7.2 (1.4–37)	0.019	5.9 (1.2–29)	0.030
LDL-cholesterol, per mmol/L	1.1 (0.8–1.4)	0.74	–	–	–	–
HDL-cholesterol, Lg10, per unit	0.7 (0.1–7.4)	0.80	–	–	–	–
S-creatinine, Lg10, per unit	108 (5.9–2005)	0.002	52.1 (1.1–2386)	0.043	14.8 (0.3–636)	0.16
Abdominal obesity	1.8 (1.0–3.5)	0.064	1.5 (0.6–3.6)	0.41	1.2 (0.5–3.2)	0.69
Hypoglycemia, severe episodes	0.4 (0.1–2.0)	0.27	–	–	–	–
Systolic BP, per mm Hg	1.10 (1.07–1.13)	< 0.001	1.07 (1.04–1.11)	< 0.001	1.08 (1.05–1.12)	< 0.001
Diastolic BP, per mm Hg	1.06 (1.02–1.09)	0.003	1.01 (0.95–1.06)	0.84	1.03 (0.97–1.08)	0.36
Depression	1.2 (0.5–2.6)	0.71	–	–	–	–
Smoking	0.5 (0.2–1.4)	0.20	–	–	–	–
Physical inactivity	0.8 (0.3–1.8)	0.57	–	–	–	–
Continuous subcutaneous insulin infusion	0.8 (0.3–2.1)	0.70	–	–	–	–
Lipid-lowering drugs	3.2 (1.9–5.4)	< 0.001	1.6 (0.8–3.3)	0.16	1.7 (0.8–3.5)	0.14
Antidepressants	1.6 (0.6–3.8)	0.32	–	–	–	–
Corticosteroids, nasal or inhaled	1.4 (0.6–3.2)	0.42	–	–	–	–
Cardiovascular complications	5.4 (1.4–21.5)	0.016	4.1 (0.8–22)	0.097	2.4 (0.5–11.1)	0.26

^a, b^ Multiple regression analyses (Backward: Wald): N = ^a^ 254/^b^ 246; Nagelkerke R Square ^a^ 0.447/ ^a^ 0.392

Hosmer and Lemeshow Test ^a^ 0.845/^b^ 0.708. For missing values, see Tables 1 and 2

## Discussion

The main findings of this study of 283 adult patients with T1D were that lower levels of EN-RAGE and higher levels of sRAGE were independently associated with the use of LLD, whereas lower levels of sRAGE were associated with the use of AHD. The use of LLD was associated with lower levels of the calculated EN-RAGE/sRAGE ratio, whereas the use of AHD was associated with higher levels of the EN-RAGE/sRAGE ratio. No other variables were associated with the EN-RAGE/sRAGE ratio.

sRAGE acts as a decoy for ligands, and blocks their interaction with RAGE and thus prevents inflammatory responses mediated by RAGE [8, 12, 14]. Higher levels of sRAGE were according to previous research associated with decreased vascular inflammation, stabilized atherosclerotic lesions, lower carotid intima-media thickness progression, and lower risk for first-time coronary events [6, 8, 15, 16]. EN-RAGE serves as a ligand of RAGE [11] and is involved in chronic inflammation in atherosclerotic lesions [10]. Causality can’t be determined by this cross-sectional study, but in previous research it was demonstrated that certain statins up-regulated sRAGE [14] and suppressed up-regulation of EN-RAGE and RAGE [19, 20]. Taking into consideration the results from previous research, it is it is plausible, but not determined, that the higher levels of sRAGE and lower levels of EN-RAGE which were linked to the use of LLD in this study are secondary to the use of LLD. In the case of AHD, it is more complicated. Low levels of sRAGE might be secondary to the underlying condition, i.e. hypertension, as essential hypertension was previously linked to lower levels of sRAGE [17]. Low levels of sRAGE might also be secondary to the use of ARBs, which has been linked to decreased levels of sRAGE [22, 23], or might be linked to other types of AHD which have not been explored in this context previously. The lower levels of sRAGE might not have any clinical significance as both lower levels of RAGE and sRAGE have been linked to ARBs [22, 23], and low levels of RAGE decrease the risk for atherosclerosis in diabetes [7]. Higher HbA1c levels have been linked to higher levels of EN-RAGE [10]. As the patients using LLD had higher levels of HbA1c, the levels of EN-RAGE would have been expected to be higher in the users of LLD which they were not. The levels of EN-RAGE were lower in the users of LLD. The findings of this study were not explained by differences in smoking habits, physical inactivity, the presence of depression, the use of antidepressants, or the use of nasal or inhaled corticosteroids, as none of these variables was associated with LLD, AHD, EN-RAGE, sRAGE, or the EN-RAGE/sRAGE ratio.

Indications for treatment with LLD were increased levels of total cholesterol and/or LDL-cholesterol and/or cardiovascular complications according to the Swedish national guidelines in 2009 [33]. As increased EN-RAGE levels have been demonstrated in T1D patients [18], and increased levels of HbA1c have been linked to higher levels of EN-RAGE in both T2D patients and non-diabetics [10], the indications for treatment with LLD should perhaps be supplemented with unsatisfactory glycemic control in patients with T1D, provided it can be further confirmed that LLD may decrease EN-RAGE levels. Alternately, if normal ranges for EN-RAGE or sRAGE would be established, these variables might be included in the decision-making regarding initiation of treatment with LLD, independent of the degree of dyslipidaemia and the degree of impaired glycemic control.

In further research it would be of interest to perform longitudinal studies, and measure levels of EN-RAGE and sRAGE before and after the introduction of LLD and AHD. It would also be of interest to compare potential effects of different types of LLD and AHD on EN-RAGE and sRAGE. Maybe additional specific drugs targeting the RAGE/sRAGE/EN-RAGE system could be developed. Another interesting research subject would be to explore potential effects of alimentary AGEs and antioxidants on the levels of sRAGE and EN-RAGE.

## Study strengths and limitations

Strengths of the study were that the inclusion and exclusion criteria were well defined. The findings of this study are new as exploration of the associations between LLD, AHD, sRAGE, EN-RAGE, and the EN-RAGE/sRAGE ratio in a clinically well-defined setting of T1D has not previously been performed. The results were controlled for relevant variables with well-known effects on cardiovascular complications and mortality. To control for nasal and inhaled corticosteroids was also relevant as an exploration showed that systemic corticosteroids were associated with the use of AHD and the EN-RAGE/sRAGE ratio. The logistic regression models were elaborated for the associations and calibrated and validated for goodness of fit with the data variables. Precise ELISA techniques were used and the ELISA assay showed a low intra-assay coefficient of variation for both sRAGE and EN-RAGE. Weaknesses were first, that no subanalyses of the antihypertensive medication were performed due to the limited sample size of T1D patients using AHD. Second, no data regarding dietary habits were provided. Third, there was no control group with persons without diabetes.

## Conclusions

In a population-based cohort of adult patients with T1D, the use of LLD was linked to higher levels of sRAGE which is an inhibitor of the inflammatory and atherogenic processes mediated by RAGE. The use of LLD was also linked to lower levels of EN-RAGE, which is a stimulator of the inflammatory processes mediated by RAGE. The study supports that LLD may have inhibitory effects on the atherogenic processes by their impact on the levels of sRAGE and EN-RAGE. In contrast, the use of AHD was linked to lower levels of sRAGE. No other variables but the use of LLD and the use of AHD were linked to the EN-RAGE/sRAGE ratio.

## Data Availability

The data set analysed during the current study is not available publicly as individual privacy could be compromised, and we have no permission from the Regional Ethical Board to share the research data publicly. The data set is available from the corresponding author on reasonable request.

## Abbreviations

ACE: Angiotensin-Converting Enzyme
AGEs: Advanced Glycation End products
ARB: Angiotensin II Receptor Blockers
AOR: Adjusted Odds Ratio
BMI: Body Mass Index
BSA: Bovine Serum Albumin
CI: confidence interval
COR: Crude Odds Ratio
CRP: C-Reactive Protein
ELISA: Enzyme-Linked Immunosorbent Assay
EN-RAGE: Extracellular Newly identified Receptor for Advanced Glycation End products
HbA1c: Hemoglobin A1c
HMG-CoA: Hydroxy-Methyl-Glutaryl Coenzyme A
HDL-cholesterol: High-density Lipoprotein-cholesterol
LDL-cholesterol: Low-density Lipoprotein-cholesterol
PBS: Phosphate-Buffered Saline
RAGE: Receptor for Advanced Glycation End products
sRAGE: soluble Receptor for Advanced Glycation End products
T1D: Type 1 Diabetes
T2D: Type 2 Diabetes
WC: Waist Circumference
